# DNA Vaccination Partially Protects against African Swine Fever Virus Lethal Challenge in the Absence of Antibodies

**DOI:** 10.1371/journal.pone.0040942

**Published:** 2012-09-26

**Authors:** Jordi M. Argilaguet, Eva Pérez-Martín, Miquel Nofrarías, Carmina Gallardo, Francesc Accensi, Anna Lacasta, Mercedes Mora, Maria Ballester, Ivan Galindo-Cardiel, Sergio López-Soria, José M. Escribano, Pedro A. Reche, Fernando Rodríguez

**Affiliations:** 1 Centre de Recerca en Sanitat Animal (CReSA), UAB-IRTA, Bellaterra, Barcelona, Spain; 2 CISA-INIA, Valdeolmos, Madrid, Spain; 3 Departamento de Biotecnología, INIA, Madrid, Spain; 4 Departamento de Microbiología I, Universidad Computense de Madrid (UCM), Madrid, Spain; 5 Departament de Sanitat I Anatomia Animals, Universitat Autònoma de Barcelona (UAB), Bellaterra, Barcelona, Spain; Virginia Polytechnic Institute and State University, United States of America

## Abstract

The lack of available vaccines against African swine fever virus (ASFV) means that the evaluation of new immunization strategies is required. Here we show that fusion of the extracellular domain of the ASFV Hemagglutinin (sHA) to p54 and p30, two immunodominant structural viral antigens, exponentially improved both the humoral and the cellular responses induced in pigs after DNA immunization. However, immunization with the resulting plasmid (pCMV-sHAPQ) did not confer protection against lethal challenge with the virulent E75 ASFV-strain. Due to the fact that CD8^+^ T-cell responses are emerging as key components for ASFV protection, we designed a new plasmid construct, pCMV-UbsHAPQ, encoding the three viral determinants above mentioned (sHA, p54 and p30) fused to ubiquitin, aiming to improve Class I antigen presentation and to enhance the CTL responses induced. As expected, immunization with pCMV-UbsHAPQ induced specific T-cell responses in the absence of antibodies and, more important, protected a proportion of immunized-pigs from lethal challenge with ASFV. In contrast with control pigs, survivor animals showed a peak of CD8^+^ T-cells at day 3 post-infection, coinciding with the absence of viremia at this time point. Finally, an *in silico* prediction of CTL peptides has allowed the identification of two SLA I-restricted 9-mer peptides within the hemagglutinin of the virus, capable of *in vitro* stimulating the specific secretion of IFNγ when using PBMCs from survivor pigs. Our results confirm the relevance of T-cell responses in protection against ASF and open new expectations for the future development of more efficient recombinant vaccines against this disease.

## Introduction

African Swine Fever (ASF) is a fatal hemorrhagic disease of domestic pigs caused by African Swine Fever Virus (ASFV), the only member of the family *Asfarviridae*, a large enveloped virus that contains a linear double-stranded DNA of 170–190 kbp encoding more than 150 proteins, many of them involved in virus-host interactions [1]. The fact that the disease is endemic in many Sub-Saharan Africa countries and Sardinia, makes this disease a threat for disease-free countries, as has been demonstrated with the recent outbreaks declared in Georgia, Russia, Armenia, and Iran [2]–[4].

Unfortunately, there is no vaccine available for ASFV. Historical attempts to protect animals with inactivated vaccines either failed or gave controversial results [5]–[7]. Studies using attenuated vaccines demonstrated their potential to protect pigs against experimental infection with homologous virulent virus but rarely, against heterologous viruses [8]–[11]. The main problem with these attenuated viral strains is due to biosafety issues, since they retained some virulence and produced sub-clinical infections in pigs, occasionally becoming chronically infected [12], [13].

Little is known about the mechanisms involved in ASF protection, and most of the experimental evidence dates from more than a decade ago. Thus, passive transfer experiments have clearly demonstrated the potential that ASFV antibodies can play in protection against experimental challenge [14], [15]. In consonance with these results, neutralizing antibodies against the structural proteins p30, p54 and p72 have been described [16]–[18]. However, pig immunization experiments using these antigens in the form of baculovirus-expressed recombinant proteins, yielded controversial results in terms of the protection afforded against the ASFV challenge [19], [20], most probably due to differences in the viral strains used.

Together with the protective potential that humoral responses can afford, new evidence has also demonstrated the role that T-cell responses can play in protection. Thus, *in vivo* depletion experiments, have demonstrated the key role of CD8^+^-T cells in the protection afforded by attenuated ASFV strains [21]. The presence of ASFV-specific cytotoxic T cells during ASFV infection had been previously demonstrated [22], also identifying p30 and p73 as potential CTL targets [23], [24]. Albeit no records exist about the protection capability of these specific CTLs *in vivo*, their induction might help to explain the partial protection afforded against ASFV after immunization with baculovirus-expressed recombinant p54 and p30 proteins [19] and overall, with the ASFV Hemagglutinin (HA) of ASFV, a protein that conferred protection in the absence of detectable neutralizing antibodies [25]. Total baculovirus-infected cell extracts were used as immunogens in all these experiments, thus increasing the chances of inducing strong CTL responses, as has been clearly stated for other viral antigens in the presence of insect-cell debris [26].

In the work presented here we add new evidence about the relevance of CD8^+^-T cell responses in protection against ASFV and demonstrate the potential of DNA immunization to design safe and efficient future vaccines against ASF. Recent evidence from our laboratory has demonstrated that DNA vaccines encoding p30 and p54 fused together (pCMV-PQ) induced good antibody responses in mice but not in pigs, where they were undetectable [27]. Here we have extended these studies demonstrating first, that DNA immunization in pigs could be exponentially improved by adding the extracellular domain of HA (sHA) to the vaccine-encoded antigens. Pigs immunized with pCMV-sHAPQ induced strong humoral and cellular responses, even though no protection was afforded against the lethal ASFV-challenge. And second that 33% of the pigs (2/6) immunized with pCMV-UbsHAPQ, encoding the same three ASFV antigens fused to ubiquitin, survived the lethal challenge. Protection was afforded in the absence of vaccine-induced antibodies and more importantly, correlated with the proliferation of antigen specific CD8^+^ T-cells recognizing two previously undescribed 9-mer epitopes, both mapping within the sHA. The implications of our results for the future development of ASFV recombinant vaccines will be profoundly discussed throughout our manuscript.

## Results

### Immunoadjuvant potential of the extracellular domain of the ASF hemagglutinin (sHA) in DNA vaccination protocols

**Figure 1:**
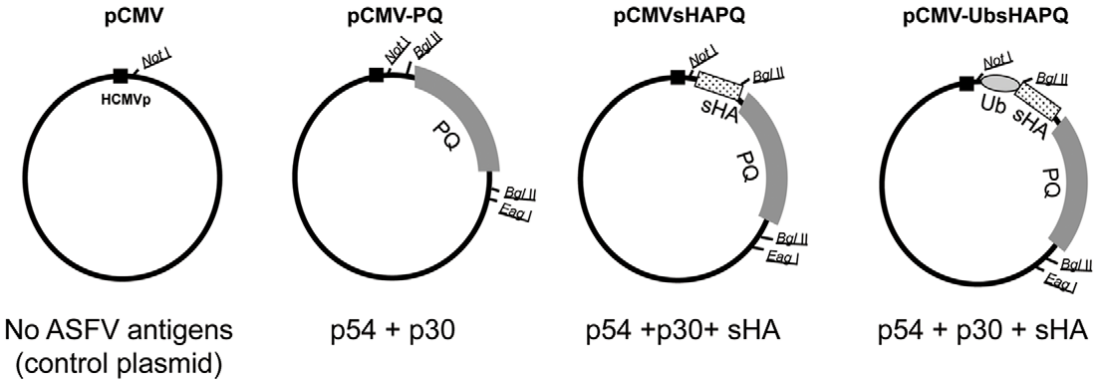
Schematic representation of the plasmids used for immunization and ASFV antigens by them encoded.

Aiming to correct the lack of protection afforded by pCMV-PQ, a DNA vaccine encoding the ASFV p54 and p30 antigens (4), the extracellular domain of the viral hemagglutinin (sHA) was fused to the N-terminal end of the p30p54 fusion construct (PQ), thus obtaining the pCMV-sHAPQ plasmid (Fig. 1). Theoretically, incorporating sHA should augment the antigenic coverage of our DNA vaccine formulation [25]. Immunofluorescence experiments allowed the detection of both p54 and p30 determinants in Vero cells transfected with pCMV-sHAPQ, showing similar levels and cellular distribution to those transfected with pCMV-PQ (data not shown).

**Figure 2:**
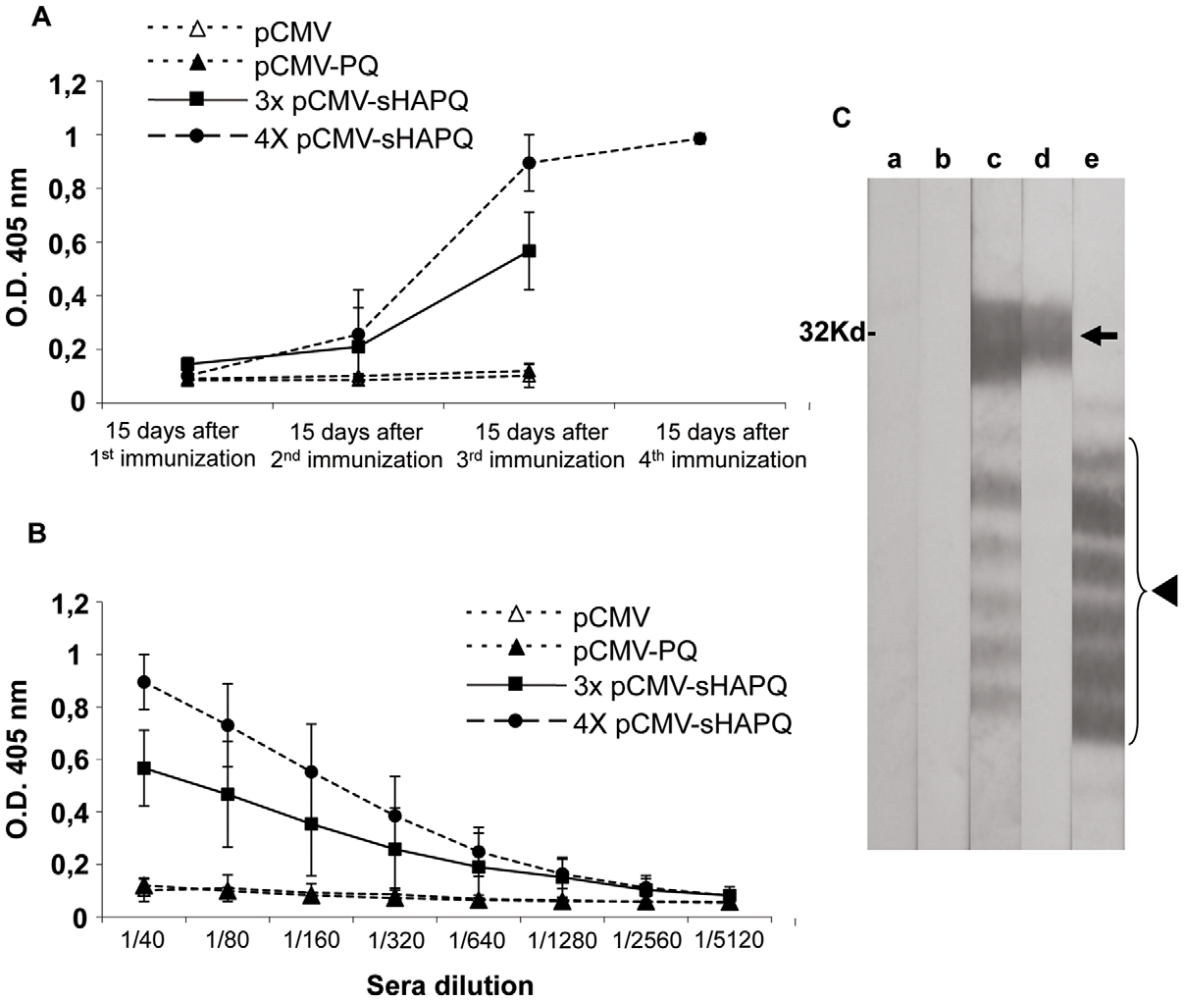
pCMV-sHAPQ induces specific antibody responses in pigs. 14 pigs were divided into three groups receiving either: the pCMV-sHAPQ plasmid (6 pigs), the pCMV-PQ plasmid (4 pigs) or the pCMV empty plasmid as controls for the assay (4 pigs). Four pigs from each group were immunized three times and two extra pigs from the pCMV-sHAPQ immunized-group received a fourth dose of this same plasmid. (A) Sera collected 15 days after each DNA vaccine administration were used to follow by ELISA the kinetics of the specific anti-p30 antibodies induced after DNA immunization. (B) Sera collected 15 days after the last DNA vaccination was used to ELISA-titrate the anti-p30 antibodies induced. Data shown correspond to average O.D values and standard deviations obtained per each immunization group. (C) Confirmatory Western-blot using ASFV infected cell extracts as antigen. Immunoreactive bands correspond to the specific recognition of the immunodominant p30 protein (arrow) and to the diverse p54 isoforms (arrow head) found in these extracts, ranging the last ones between 22 and 27 KD [65], [66]. Figure shown corresponds to the results obtained with a representative serum (1∶100 dilution) obtained 15 days after the third immunization with pCMV (a), pCMV-PQ (b) or pCMV-sHAPQ (c). The anti-p30 monoclonal antibody (d) and the specific polyclonal anti-p54 antibody (e) confirm the specificity of the reactions.

Once the in vitro expression of both plasmids was confirmed, an initial in vivo experiment was performed in pigs. Six Landrace X Large white pigs were immunized with pCMV-sHAPQ while for comparative analysis a group of 4 pigs were immunized with pCMV-PQ. An additional group of 4 animals received pCMV as negative controls for the assay. Four animals from each group received three DNA-shots at 15-day intervals, and two additional pigs from the pCMV-sHAPQ group received one extra dose of DNA (a total of four doses). All fourteen pigs were bled 15-days after each injection to analyze the specific antibodies induced by ELISA and/or western-blotting. In contrast with the lack of response induced by pCMV and pCMV-PQ, every single pig immunized with pCMV-sHAPQ showed detectable specific antibody responses against p30, reaching a plateau after the third injection (Fig. 2A). An additional boost (fourth DNA injection) did not seem to improve the level of antibodies induced (Fig. 2B). Specific antibodies against p54 were also detectable in the serum of each pig immunized with pCMV-sHAPQ, following exactly the same kinetics as the anti-p30 antibodies and reaching their maximum level after the third immunization, as shown by western-blotting using ASFV extracts (Fig. 2C). No responses were detectable against HA (data not shown).

**Figure 3:**
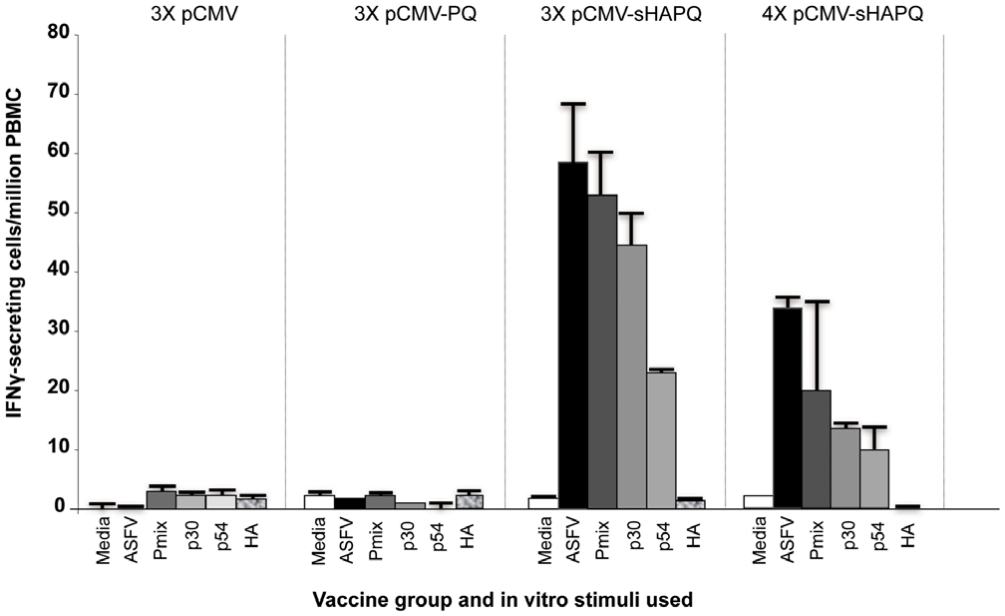
pCMV-sHAPQ induces specific T-cell responses in pigs. PBMCs obtained before ASFV challenge from pigs immunized with pCMV, pCMV-PQ or pCMV-sHAPQ (immunized three or four times), were *in vitro* stimulated with media (negative control), with the E75 ASFV isolate (10^5^ HAU_50_/ml), with a mix of the p54, p30 and HA ASFV proteins (6 μg/ml of each one) or with each one of these proteins individually. Values shown correspond to the average number of IFNγ-secretory cells detected per million of PBMCs. Standard deviations found within each group are also represented.

Immunization with pCMV-sHAPQ also induced specific T-cell responses in every single pig, detectable by IFNγ-ELISPOT after in vitro stimulation with either live-ASFV or with the specific recombinant proteins. PBMCs obtained 15 days after each immunization with pCMV-sHAPQ showed detectable IFNγ-secretory T-cells after in vitro stimulation with ASFV (Fig. 3). As occurred for the antibody responses, no responses were detectable against HA, the specificity of the T-cell responses being limited to p30 and p54 (Fig. 3). As with the humoral responses, no boosting effect was observed for the T-cell responses induced between the third and the fourth vaccine dose (Fig. 3). The lack of responses observed with pCMV-PQ confirmed recent results obtained in our laboratory [27], demonstrating the potential adjuvant properties of the ASFV sHA.

### pCMV-sHAPQ does not protect pigs from ASFV lethal challenge

To evaluate the protective potential of pCMV-sHAPQ, the four pigs immunized with three doses of this plasmid (pig numbers 9 to 12), were infected with 10^4^ UHA_50_ of the E75 virulent strain. The groups of pigs immunized with either pCMV or pCMV-PQ were used as controls for the assay. Clinical signs of ASF were monitored daily and pigs were bled every other day to follow viremia.

**Figure 4:**
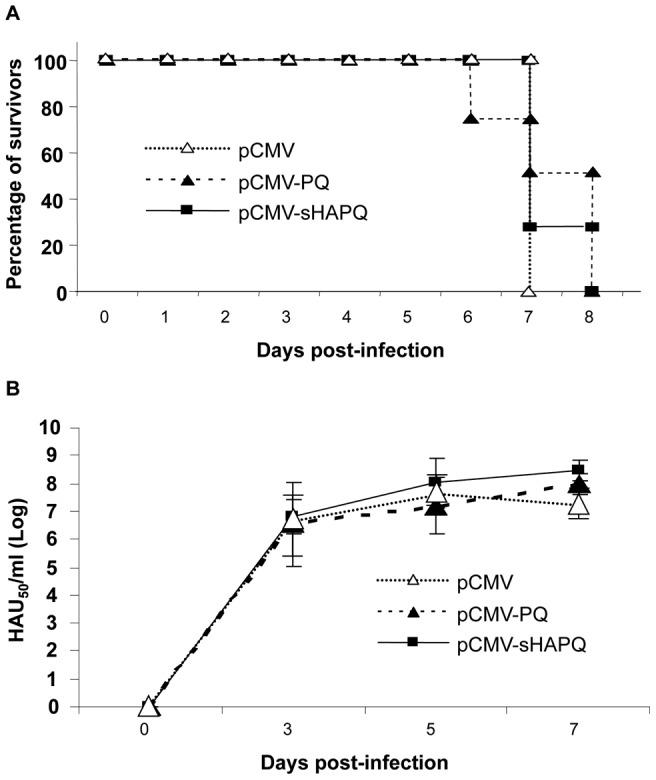
pCMV-sHAPQ does not protect against lethal ASFV challenge. (A) Surviving kinetics of pCMV, pCMV-pCMV-PQ and pCMV-sHAPQ immunized pigs after lethal challenge with ASFV (10^4^ UHA of the E75 isolate). (B) Viremia kinetics of these same individuals after ASFV challenge (days 3, 5 and 7 post infection). Results are represented as the logarithm of HAU_50_/ml serum (mean and standard deviation from each group are shown).

Despite the good humoral and cellular responses induced after vaccination with pCMV-sHAPQ, immunized animals showed similar ASF clinical signs to pCMV and pCMV-PQ pigs, all dying between days 6 and 8 post-infection (p.i.) (Fig. 4A), showing post-mortem lesions typical of acute ASFV infections indistinguishable from the other groups. In agreement with these findings, the three groups of pigs showed similar viremia kinetics and almost identical virus titres at each time-point p.i. tested (Fig. 4B) in two separate experiments. Lack of protection correlated with the inability of the sera from the pCMV-sHAPQ immunized pigs to neutralize ASFV infection (data not shown).

### DNA vaccination “a la carte”: avoiding the humoral response

To avoid the induction of antibody responses, we decided to design a new plasmid, encoding the three ASFV antigens: p54, p30 and sHA, fused to ubiquitin (pCMV-UbsHAPQ; Fig. 1); a strategy designed to enhance the CTL responses while avoiding the induction of antibodies. In contrast to the correct in vitro expression of the antigens encoded by either pCMV-PQ or pCMV-sHAPQ, no expression was detected after transfection with pCMV-UbsHAPQ, unless cells were treated with the MG132 proteasome inhibitor (data not shown). Once the in vitro targeting of the ubiquitinated construct to the proteasome and its efficient degradation were demonstrated, a second set of in vivo experiments was performed. On this occasion, twelve Landrace X Large white pigs were immunized with pCMV-UbsHAPQ and four extra-pigs were immunized with the empty plasmid (pCMV) as controls for the assay. Half of the animals of each group (six pigs with pCMV-UbsHAPQ and two with pCMV) were immunized twice while the other half were immunized four times with the corresponding plasmids.

As expected, pigs immunized with pCMV-UbsHAPQ showed no detectable antibody responses against either p30 or p54 (data not shown). The lack of antibody induction might represent the *in vivo* reflection of the successful degradation of the ubiquitinated ASFV proteins, as has previously been described for other antigens [28].

**Figure 5:**
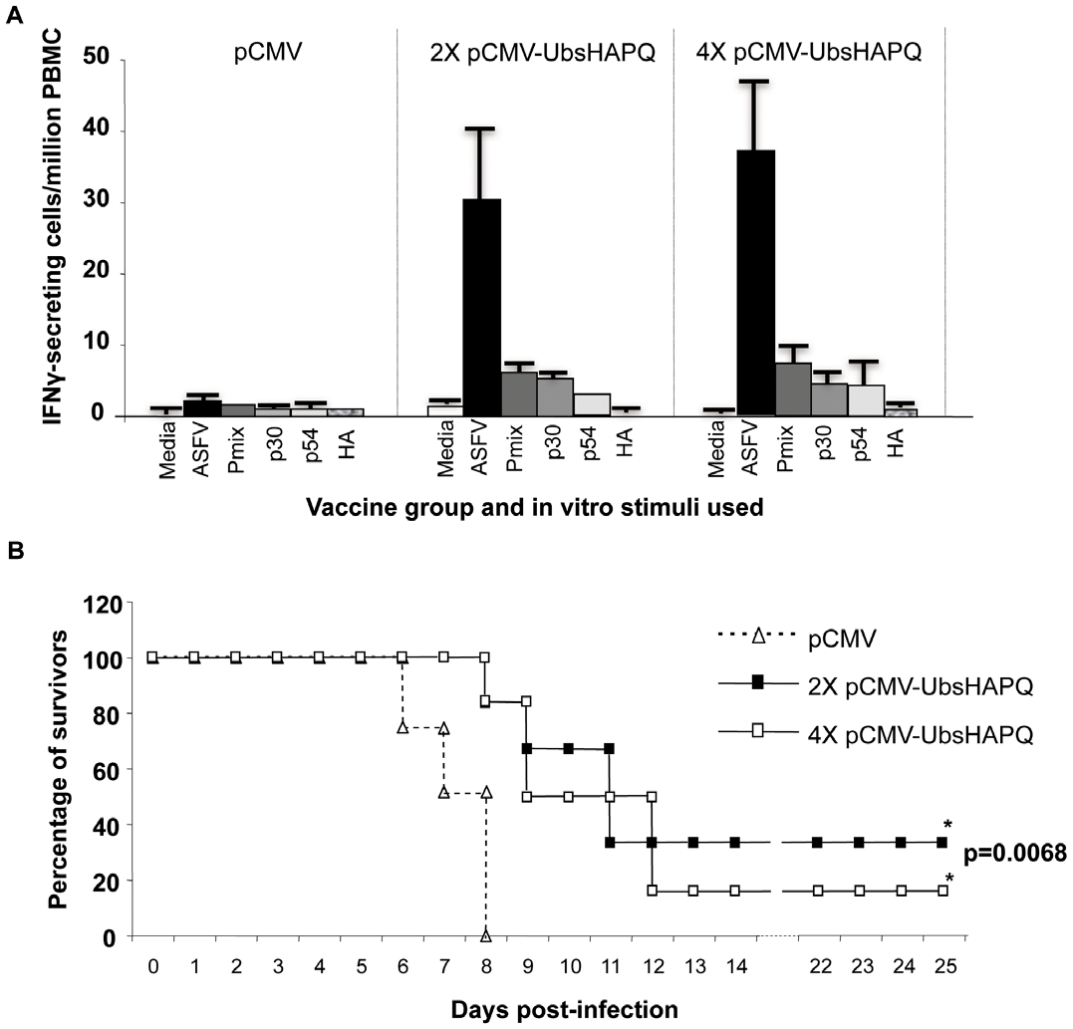
pCMV-UbsHAPQ protects against lethal ASFV challenge. (A) PBMCs from pigs immunized twice (2x) or 4 times (4x) with pCMV-UbsHAPQ or with pCMV, were *in vitro* stimulated with media (negative control), with the E75 ASFV isolate (10^5^ HAU_50_/ml), with a mix of the p54, p30 and HA ASFV proteins (6 μg/ml of each one) or with each one of these proteins individually. Values shown for each immunization group correspond to the average number of specific IFNγ-secretory cells detected per million PBMCs. Standard deviations found within each group are also represented. (B) Immunized pigs were challenged with a lethal dose of ASFV (10^4^ UHA of the E75 isolate) and survival record were plotted. Groups immunized with pCMV-UbsHAPQ showed statistically significant differences (*) when compared with the control group (p = 0.0068).

Albeit qualitatively similar, the magnitude of the IFNγresponsesinduced by pCMV-UbsHAPQ seemed to be lower by ELISPOT than those obtained with pCMV-sHAPQ. This was particularly evident after in vitro stimulation with a mixture of the recombinant proteins or individually with the specific p30 and p54 proteins (compare figures 3 and 5A). As described for the pCMV-sHAPQ immunized animals, no specific stimulation was found in response to the recombinant HA protein (Fig. 5A). However, all pigs showed relatively high levels of specific T-cells capable of secreting IFNγin response to in vitro stimulation with ASFV (Fig. 5A). As had occurred with pCMV-sHAPQ, no boosting effect was evident.

### pCMV-UbsHAPQ confers partial protection against ASFV challenge

**Figure 6:**
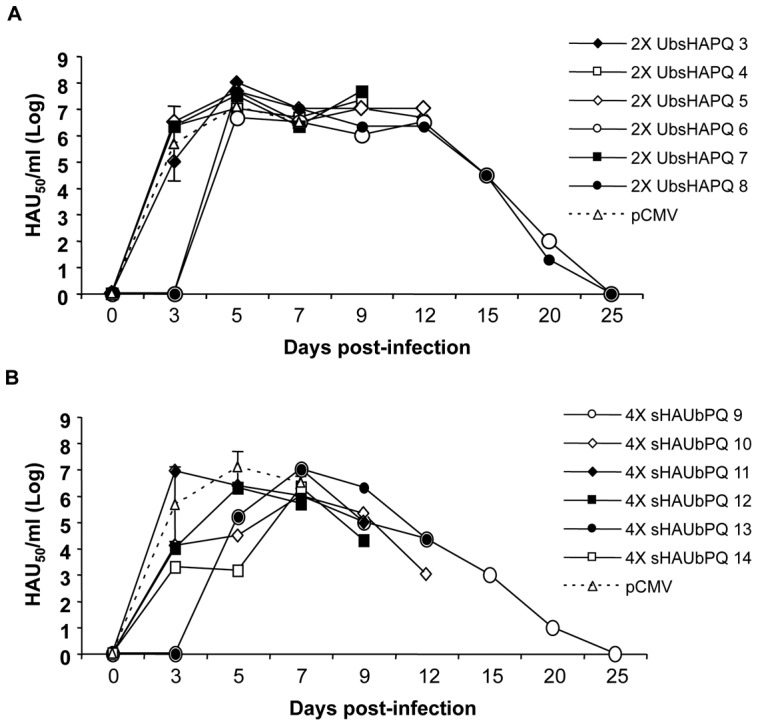
Surviving pigs show a delay in the viremia onset and clear the virus by day 25 post-infection. Pigs immunized with pCMV-UbsHAPQ, either twice (A) or four times (B) were infected with 10^4^ UHA of the E75 isolate and blood samples were taken serially at days 3, 5, 7, 9, 12, 15, 20 and 25 to follow the viremia kinetics. Individual results are represented as the logarithm of HAU_50_/ml serum, while mean and standard deviation values are represented in both panels for the pCMV-immunized control group.

In contrast to the rest of the DNA-constructs tested to date in our laboratory, a proportion of the pigs immunized with pCMV-UbsHAPQ survived the lethal challenge with ASFV. A total of three pigs survived the lethal challenge: 2 out of 6 (33%) immunized twice with pCMV-UbsHAPQ and 1 out of 6 in the group receiving four plasmid doses (Fig. 5B). As expected, control animals (pCMV group) died between days 7 and 8 post-challenge, following the typical ASF course, while in clear contrast, only 2 out of 12 pigs, immunized with pCMV-UbsHAPQ (one per group) died at day 8pi. By day 10 p.i. more than 50% of the pigs immunized with pCMV-UbsHAPQ were still alive (a fact never observed in naïve pigs challenged with this viral dose) and by day 12, the general body condition of surviving pigs had rapidly improved, including fever (data not shown). By the end of the experiment, at day 25 p.i. survivor pigs were totally recovered, not showing clinical signs of ASF, nor viremia. Confirming these results, no virus was detectable in any of the tissues tested, including spleen, tonsils and retropharingeal lymphnodes. As expected, high viral loads (ranging between 106 and 108 HAU50/gr of tissue) were found in those same organs in control animals at the time of necropsy. Interestingly, surviving pigs showed no virus at day 3 p.i., a time at which the rest of the animals showed high viremia titres (Fig. 6). The in vivo significance of the survival results was statistically confirmed. Thus, both groups immunized with pCMV-UbsHAPQ showed statistically significant differences when compared with the control group (p = 0.0068), while no significant differences were found between the groups receiving two or four doses of the pCMV-UbsHAPQ plasmid (p = 0.7418).

Confirming the absence of specific B-cell priming, pCMV-UbsHAPQ-immunized pigs did not show any detectable boosting effect on the specific antibody responses induced after ASFV challenge, showing indistinguishable antibody-kinetics from control animals after ASFV infection (not shown).

### Protection correlates with the expansion of ASFV-specific CD8^+^ T-cells: identification of two immunodominant epitopes within the ASFV HA

**Figure 7:**
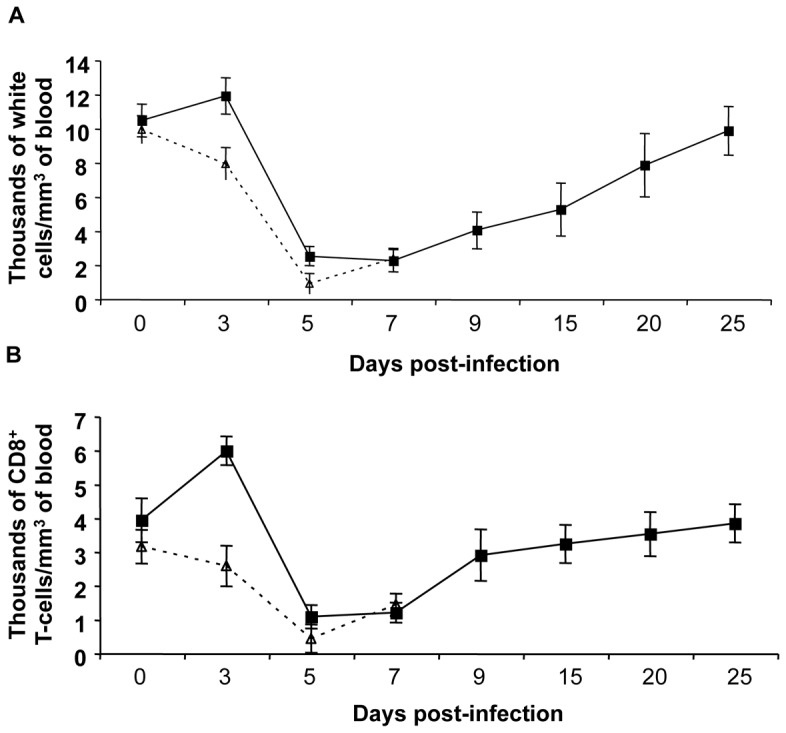
Surviving pigs show a rapid recovery from the ASFV-provoked leukopenia and a dramatic expansion of CD8^+^ T-cells. (A) Total nucleated-cell counting using whole blood samples taken at different days after ASFV challenge. (B) Number of CD8^+^ T-cells found at different times post-infection from these same blood samples after PBMC separation and CD8^+^ surface specific staining using the specific anti-CD8 monoclonal antibody. Values represented in both panels correspond to the average number of cell per mm^3^ of pig blood. Standard deviation shown corresponds to those found within the pCMV-control group (dashed line) and those found for the three surviving pigs, pre-immunized pCMV-UbsHAPQ (solid line). No significant differences were observed in the cell counts (nor in the total, nor the specific CD8^+^ T-cell counts), between non-surviving pigs, independent of the DNA plasmid used.

As represented in Figure 7A, control animals showed the characteristic ASFV-leukopenia as soon as three days after challenge, with the number of cells in blood dropping below 10^6^ cells/ml from day 5 after infection, they were incapable of recovery and finally died between days 7 and 8 p.i. In contrast, surviving pigs showed a clear delay in the appearance of leukopenia, even showing a higher number of cells in blood by day 3 p.i. (Fig. 7A), coinciding with the lack of virus at this time point (Fig. 6). Surviving pigs, however, suffered a dramatic drop in the total number of cells by day 5 p.i., initiating their recovery and finally stabilizing cell counts (Fig. 7A). Cell surface staining showed almost identical kinetics for the CD8^+^ T-cell subset (Fig. 7B). None of the rest of the cell subsets analysed (CD4^+^ T-cells, SW172a^+^ cells or CD163^+^ cell) showed such significant variations in their cell numbers during the ASFV infection (data not shown), therefore allowing us to focus our work on identifying the specificity of the expanded CD8+ T-cells.

**Figure 8:**
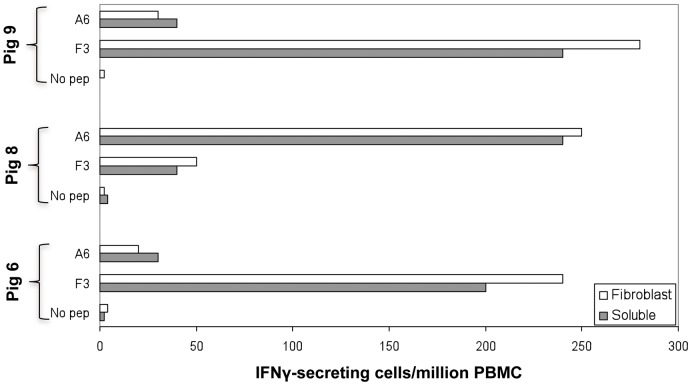
Identification of protective SLAI restricted 9-mer T-cell peptides. PBMCs from surviving pigs, pre-immunized with pCMV-UbsHAPQ (obtained at day 25 after ASFV lethal challenge), were *in vitro* stimulated with 53 9-mer peptides predicted as potential CTL epitopes. Data represented in grey boxes corresponds to the specific responses found for the two individual sHA epitopes: F3, and A6. Data represented in open boxes corresponds to the values obtained after *in vitro* stimulation with peptide-coated autologous SLAI^+^/SLAII^−^ skin fibroblasts.

These findings encouraged us to focus our efforts towards the theoretical prediction of CD8+ T-peptides within the vaccine-encoded antigens. A panel of 53 9-mer peptides selected in silico from within the p54 (5), p30 (18) and sHA (30) protein sequences, according to their theoretical potential as CTL epitopes (details in Materials and Methods), were tested by IFNγ-ELISPOT. From all the peptides tested, only two were capable of specifically stimulating PBMCs from survivor pigs to secrete IFNγ, both belonging to the ASFV HA: the F3 peptide (SVDSPTITY; positions 116 to 124 of HA) and the A6 peptide (TNGDILNYY; positions 155 to 163 of HA). An immunodominance hierarchy seemed to exist in the responses induced. Two of the three survivor pigs (pigs 6 and 9) mainly responded against the F3 peptide and more weakly against the A6 peptide, while the third survivor showed dominant responses against the A6 peptide and subdominant responses against the F3 peptides (Fig. 8). Confirming their immunostimulatory capability, SLAI^+^/SLAII^−^ skin fibroblasts derived from surviving pigs were very efficient at presenting F3 and A6 peptides to their autologous PBMCs (Fig. 8).

## Discussion

Fifty years after first entering the Iberian peninsula from Western Africa [29], ASFV has emerged again as real threat both for European and Asian countries, due to the ASFV outbreaks first declared in Georgia 2007 [2], this time imported from Eastern Africa [3], [30], [31]. In spite of the relatively profound knowledge that exists about some relevant aspects of ASFV biology, there is no vaccine available, and no effective treatment against ASF. Therefore, the control of ASF relies on its efficient diagnosis and the subsequent culling of infected animals, measures unadoptable in poor endemic African countries.

Little is known about the immunological mechanisms involved in protection against this complex virus, and even less about potential vaccine targets. Since original attempts of vaccination against ASFV, based on live attenuated and inactivated viruses were not very satisfactory [5], [12], few vaccine prototypes have been experimentally tested in vivo. Targeted disruption of specific genes such as those encoding proteins involved in evading host defence might yield promising results for the future development of safer and efficient recombinant-live attenuated vaccines. Despite the fact that this technology has been successfully used to characterize relevant ASFV virulence factors *in vivo* [32]–[37], to date no further evidence has been presented about the use of recombinant viruses obtained for vaccine purposes, most probably due to the residual virulence they retain [12], [13]. Besides these attempts, few approaches have been used to identify potential targets to develop subunit vaccines, most have been based on their antigenic properties that have not however succeeded in inducing protection [38] or have induced partial protection against ASFV challenge [25], [39]. Our first attempts to induce a protective immune response against ASFV with DNA vaccines encoding two of these antigens (p54 and p30) in the form of the chimeric protein (PQ) totally failed [27]. Here, we have extended these studies by incorporating the extracellular domain of the ASFV haemaglutinin (sHA) to our vaccine formulation, aiming to augment the antigenic coverage of our DNA vaccine [25]. Addition of sHA dramatically improved both the humoral and the cellular responses induced against the fused antigens: p54 and p30, reaching optimal results after three intramuscular injections. The sequence similarity between sHA and the CD2 leukocyte molecule [40], [41] allowed speculation about its capability to target the antigens to cells expressing the CD2 receptors CD48 and CD58 [42]–[44], such as Antigen Presenting Cells (APCs). Therefore, the improvement of the immune responses induced by the sHA fusion could be the result of antigens targeting to the APCs. Antigen targeting has proven to be successful in many other systems [45], [46], and similar results were obtained by targeting these ASFV antigens with a single chain antibody (ScFv) recognizing the SLA II molecules [27]. Further work should be done to definitively demonstrate if fusion of sHA to antigens results in targeting to cells expressing the CD2 ligands, and the relevance of such targeting in the immune responses induced. The improvement of the immune responses induced by incorporating sHA might also be due to the potential presence of T-helper cell epitopes in this molecule. Unfortunately enough, in vitro stimulation with the full-length HA did not allow confirming this hypothesis. Further work should be done in the future on this direction.

Despite the good immune response obtained after immunization with pCMV-sHAPQ, pigs were not protected from lethal challenge, showing undistinguishable ASF clinical signs and viremia kinetics from control animals. Lack of protection coincided with the induction of specific antibodies that, however, did not neutralize ASFV infection *in vitro*, an activity which had been previously associated with *in vivo* protection [19], [47]. This data seems to confirm very recent results obtained in our laboratory, showing the inability of DNA vaccines to induce neutralizing antibodies against ASFV p30 and p54 proteins that conversely, might even exacerbate ASFV infection [27]. Besides the potential adjuvant effect of sHA, currently being explored to improve the immune responses induced against other antigens in pigs (PCT/ES2008/000264), the results obtained after immunization with pCMV-sHAPQ were by itself extremely informative from the scientific point of view, as later demonstrated with the protection afforded with pCMV-UbsHAPQ, a new plasmid construct encoding exactly the same ASFV determinants.

Aiming to avoid the induction of undesired antibodies and to improve the specific CD8-T cell responses, a new vaccine was designed, pCMV-UbsHAPQ, encoding exactly the same antigenic determinants: p54, p30 and sHA, fused to the cellular polypeptide ubiquitin. As expected [28], vaccination with pCMV-UbsHAPQ did not induced antibody responses in pigs but conferred partial protection against ASFV challenge, confirming the relevance of T-cell responses in protection against ASFV. Our approach, far from being obvious, allowed the dissection of the role that CD8^+^ T-cell responses could play in the protection afforded by our DNA vaccine against ASFV in its domestic host, an almost neglected issue in the specialized literature. The work done by Oura et al (41) is in our understanding, the only study clearly demonstrating the relevance of CD8^+^ T-cells in the protection afforded by attenuated ASFV viruses, albeit the specificity of such CD8^+^ T-cells has never been described. Our results not only confirm and extend these studies to the field of DNA vaccines but also identify for the first time, some of these potentially protective CD8^+^ T-cell epitopes from within the sHA ASFV antigen. The protection afforded was not improved by increasing the number of DNA-vaccine doses, probably reflecting the lack of boosting effect for the T-cell responses induced after one boost. Conversely, we found that 2 out of 6 pigs that received 2x doses of pCMV-UbsHAPQ survived (33%) while only 16% of the pigs that received 4x plasmid doses survived. Although not statistically significant, the same results were obtained in two separate experiments, indicating that multiple boosting could be somehow deleterious in terms of the protection afforded. Of the possible factors contributing to the inconsistencies in the relatively low protection conferred by this vaccine, we hypothesize that immunization with 4x doses of pCMV-UbsHAPQ might be able to induce low levels of non-neutralizing and exacerbating antibodies [27] that in turn, could counteract the protective effects of the specific CD8-T cells induced by the vaccine. Albeit undetectable by our methodology, we should not rule-out this possibility. Previous results obtained in the lab using the mouse model seemed to confirm this hypothesis since a few mice immunized with 4x dosed of pCMV-sHAPQ developed low, albeit significant specific antibody levels. Additionally, prime-boost regimes priming with 2x doses of pCMV-UbsHAPQ and boosting with heterologous expression systems encoding the full length sHAPQ (without ubiquitin), did not confer protection against ASF-challenge, correlating with the induction of non-neutralizing antibodies (unpublished results).

Despite the fact that pCMV-UbsHAPQ induced qualitatively similar T-cell responses to pCMV-sHAPQ after in vitro stimulation with ASFV, p54 or p30 (no responses were ever detected after stimulation with HA), the magnitude of these responses seemed overall to be quantitatively lower for the former when using the recombinant proteins (compare figures 3 and 5 A), most probably reflecting an immunological bias of the immune responses induced by the ubiquitinated construct towards the induction of peptide-specific CD8^+^ T-cell responses. This theory was definitively confirmed by demonstrating the potential of the PBMCs from pigs that survived after pCMV-UbsHAPQ immunization, to efficiently secrete IFNγ after *in vitro* stimulation either with the synthetic soluble peptides (F3 and A6) or with autologous peptide-coated fibroblasts. Lack of available specific peptides at the time of performing the in vivo experiment 1 did not allow the performance of in vitro-peptide stimulation experiments with PBMCs from pCMV-sHAPQ pigs. However, today we know that the main source of IFNγexpressed by PBMCs from pigs immunized with pCMV-sHAPQ was a subset of specific CD4^+^ T-cells that specifically proliferated in response to 5-day-stimulation with p30 and p54 protein but did not respond to the addition of the 9-mer sHA peptides. Conversely, a specific expansion of CD8^+^ T-cells was observed when stimulating PBMCs from the pCMV-UbsHAPQ immunized pigs with the specific sHA peptides, confirming their SLAI restriction (manuscript in preparation).

Interestingly, the two SLA I-restricted epitopes identified, from the 53 predicted in silico, mapped within the sHA. The fact that none of them mapped within the p54, nor the p30, a protein previously described as a potential CTL target [24], might help to explain the lack of protection afforded by DNA vaccines encoding exclusively p54 and p30 [27]. Confirming this theory, the non-protective pCMV-sHAPQ plasmid was only capable of inducing responses against p54 and p30, while no responses were detectable against HA, nor the sHA peptides (data not shown).

Prediction of peptide-MHC I binding is the main basis for the anticipation of CTL epitopes [48], [49]. However, polymorphism of pig MHC I molecules (SLA I) with few known SLA I restricted epitopes hinders the prediction of CTL epitopes by their binding to SLA I. Therefore, we used an alternative approach based on their binding affinity to the transporters associated with antigen processing (TAPs), molecules which shuttle the peptides from the cytosol to the endoplasmic reticulum to be presented by MHCI molecules. Although the TAP-binding affinity model used here was initially designed for human predictions [50], [51], computational evidence suggests that the model could be applied to mammals in general. Lack of detectable specific IFNγ-responses after PBMCs stimulation with any of the other 51 synthesized peptides might highlight: limitations in our theoretical predictions, technical limitations of our read-out assays, the establishment of immunodominance hierarchies masking subdominant responses or the absence of other CTL epitopes with protective potential within p54, p30 and sHA, at least to be presented in the SLA I context of the pigs used in this study. Peptide immunization experiments using either conventional pigs or specific pathogen free (SPF) pigs (manuscript in preparation), seems to confirm the protective potential of the F3 and A6 CTL peptides in a proportion of pigs that varied between 20–33% of the animals, pointing toward a SLA I-restricted protection. We are currently haplotyping the surviving pigs from these and other experiments being performed with other viral determinants aiming to confirm this hypothesis; a long term objective, since haplotyping domestic pigs it is not an easy task, even for laboratories exclusively working on this issue.

In summary, the results presented in this work demonstrate not only the potential of DNA immunization as a strategy to design future ASFV vaccine candidates, but also confirm the importance of the vaccine-induced CD8^+^ T-cell responses against ASFV and identifies for the first time two SLA I-restricted CD8^+^ T-cell epitopes with putative protective potential. We believe that the future development of an effective vaccine against such a complex virus will need the induction of both, broader CTL responses covering the SLA I-heterogeneous swine population and neutralizing antibodies. We are currently addressing these goals following two different approaches: on one hand, we are attempting to identify as many CTL epitopes as possible within the ASFV polypeptides by using ELI libraries [52] developed in the laboratory from the entire ASFV genome (manuscript in preparation) and on the other hand, we are attempting to obtain broader and optimal cellular and humoral responses concomitantly by testing several strategies of proved efficacy also in Veterinary Medicine, including: electroporation to improve DNA vaccine delivery [53]; the use of co-stimulatory molecules such as Synthetic oligodeoxynucleotides (ODNs) containing unmethylated CpG motifs [54]; and “prime-boosting” strategies [55], [56].

## Materials and Methods

### Ethics Statement

All experiments were performed in the Biosafety Level 3 facilities of the Centre de Recerca en Sanitat Animal (CReSA-Barcelona). Animal care and procedures were performed in accordance with the guidelines of the Good Experimental Practices (GEP) and under the supervision of the Ethical and Animal Welfare Committee of the Universitat Autònoma de Barcelona (UAB; Permit Number: DMAH-5796). Animals were observed daily according to a welfare schedule in order to monitor their health status, and check list (approved by the CRESA Animal Welfare Committee) to monitor the clinical aspects after the infection with ASFV.

### Plasmid construction

The open reading frame (ORF) encoding the extracellular domain of the ASFV hemagglutinin (sHA) from the Spanish isolate E75 was obtained by PCR using the forward (5′-GCGGCCGCCATGTGGAGTACTTTAAATCAAAC-3′) and reverse (5′-CGGCCGAGATCTTGTGGATAAATAATTTTG-3′) primers and the pBakpak-HA plasmid [25] as template. The amplified fragment was cloned in the unique *Not*I cloning site of pCMV plasmid (Clontech) to obtain pCMV-sHA with a *Bgl*II unique site at the 3′-end for further cloning purposes.

The ORF encoding PQ, a chimeric fusion of the ASFV p30 and p54, also from the Spanish isolate E75 was PCR amplified using the forward (5′-GGATCCATGGATTCTGAATTTTTTCAACCGG-3′) and reverse (5′-AGATCTTACAAGGAGTTTTCTAGGTC-3′) primers and the pCMV-PQ plasmid [27], as template. The amplified fragment was digested with the *BamH*I and *Bgl*II restriction enzymes and cloned into the unique *Bgl*II cloning site of pCMV-sHA to obtain the pCMV-sHAPQ plasmid, encoding PQ in frame with the ASFV sHA polypeptide.

Finally, the ORF encoding sHAPQ was PCR amplified using the forward (5′-GGATCCATGTGGAGTACTTTAAATCAAAC-3′) and reverse (5′-AGATCTTACAAGGAGTTTTCTAGGTC-3′) primers containing the *Bam*HI and *Bgl*II restriction sites and the newly generated pCMV-sHAPQ plasmid as PCR-template. The amplified fragment was cloned using the *Bgl*II site of pCMV F1/2 Ubiq plasmid [57], to obtain the plasmid pCMV-UbsHAPQ, encoding sHAPQ fused to cellular ubiquitin.

All the products were expressed under the control of the immediate early promoter of human cytomegalovirus.

A schematic representation of the plasmids used throughout this work is provided (Fig. 1). In order to facilitate the comprehension of the results obtained, the specific ASFV products encoded by each vaccine (if any) are also indicated.

### Immunization of animals and in vivo experimental design

Experiments with 8-week-old Landrace X Large white pigs were always performed under the approval of the Ethical and Animal Welfare Committee of the Universitat Autònoma de Barcelona and infections with ASFV were done under BioSecurity level 3 (BSL-3) conditions at CReSA. DNA immunization experiments (experiments 1 and 2) were performed using different numbers of doses (600 μg of DNA each) of the corresponding endotoxin-free DNA plasmid (Qiagen), administered at 15 day intervals. One third of each vaccine dose was injected in the rectus femoris quadriceps muscle, one third in the trapezius muscle of the neck and the last third was subcutaneously injected in the ear. Experiments were repeated twice to ensure the reproducibility of the results.

#### In vivo experiment 1

14 pigs were divided into three groups receiving either: the pCMV-sHAPQ plasmid (6 pigs), the pCMV-PQ plasmid (4 pigs) or the pCMV empty plasmid as controls for the assay (4 pigs).

#### In vivo experiment 2

16 pigs were divided into three groups receiving either: the control pCMV plasmid (4 pigs), the pCMV-UbsHAPQ plasmid twice (6 pigs) or the pCMV-UbsHAPQ plasmid four times (6 pigs).

### Antibody detection

ASFV specific antibodies in pig sera were detected by conventional ELISA assay to detect specific antibodies against either whole ASFV or the p30 structural protein, as previously described [58]. To confirm the positive results, sera were tested by western blot assay using crude protein extracts obtained either from *Thriclopusia ni* larvae infected with a recombinant baculovirus expressing the ASFV p54 protein [59], the ASFV HA [25] or from ASFV infected Vero cell supernatants processed as previously described [60]. Samples were run on 4–20% Tris-glycine SDS-polyacrylamide gels (Novex) followed by transfer to nitrocellulose filters (Bio-Rad). Next, membranes were cut and divided into 4 mm wide strips, blocked with 2% non-fat milk and then tested with individual sera diluted 1∶100 in PBS-milk. Finally, the presence of immunocomplexes was detected using either a peroxidase-labeled goat anti-mouse IgG antibody or a peroxidase-labeled mouse anti-pig IgG antibody (Sigma). Colorimetric detection was achieved by using 4-Chloronaphtol as substrate (Sigma). Albeit antibodies against p30 and p54 are detectable both by ELISA and by Western blotting, the sensitivity of the techniques change for each one of these ASFV antigens, the latter being ideal to specifically evaluate the induction of anti-p54 antibodies [59].

The detection of neutralizing antibodies was performed as previously described [61]. Briefly, 10 fold serial dilutions of the E75 ASFV strain (starting with 10^5^ hemadsorbing units (HAU_50_), were mixed (1∶2) with the corresponding inactivated pig serum. After a 1 h incubation at 37°C in a 96-well plate, 4×10^5^ pulmonary alveolar macrophages (PAMs) were added to each well and cultured for 24 hours, before adding porcine erythrocytes (diluted 1/100). To follow the appearance of hemadsorption, plates were observed every 12 hours. As a control for the assay, hyperimmune serum from a pig that had recovered from an ASFV infection was used.

### Theoretical prediction of CTL epitopes

In silico prediction of CTL epitopes was based on their binding affinity to TAP using a support vector machine regression model [50] that was extended to account for the fact that MHCI-restricted epitopes can often be transported by TAP as N-terminally elongated peptide precursors [51]. Following this method, we designed 53 9-mer peptides from the antigens encoded within pCMV-UbsHAPQ, corresponding with the ASFV proteins: p54, p30 and sHA. These peptides were individually used as stimulators in IFNγ-ELISPOT assays.

### Swine IFNγ-ELISPOT

Peripheral blood mononuclear cells (PBMC) were separated from whole blood by density-gradient centrifugation with Histopaque 1077 (Sigma). For PBMC cultures, RPMI 1640 medium supplemented with 10% foetal calf serum (Invitrogen), 50,000I U penicillin/l (Invitrogen) and 50 mg streptomycin/l (Invitrogen) was used. Trypan blue was used to assess cell-viability. The frequency of ASFV-specific IFNγ-secreting cells (IFNγ-SC) in PBMC were analysed by an ELISPOT assay using commercial mAbs (Swine IFNγ Cytoset; Biosource Europe) following a previously reported method [62]. Briefly, 96-well plates (Costar 3590; Corning) were coated overnight with 8.3 µg/ml of IFNγ capture antibody diluted in carbonate/bicarbonate buffer (pH9.6). Plates were then washed and blocked for 1 h at 37°C with 150 µl PBS with 0.5% BSA. Once the blocking solution was eliminated, 5×10^5^ PBMCs were dispensed per well and cultured with the appropriate stimulus: ASFV E75 isolate was used at 10^6^ HAU_50_/ml, baculovirus-expressed p30, p54 and HA proteins were used at 6 μg/ml (a baculovirus expressing an irrelevant protein was used as control), and peptides from p30, p54 and HA proteins were used at 1 μg/ml. After a 20 h incubation at 37°C in a 5% CO_2_ atmosphere, cells were removed and the biotinylated detection antibody was added at 2.5 µg/ml and incubated for 1 h at 37°C. The reaction was revealed by sequential incubation of plates with streptavidin–peroxidase (1 h) and insoluble 3,3′,5,5′-Tetramethylbenzidine (TMB; Calbiochem). Unstimulated cells and phytohaemagglutinin (PHA)-stimulated controls (5 µg/ml) were also included. To calculate the ASFV-specific frequencies of IFNγ-secreting cells, counts of spots in unstimulated wells were subtracted from counts in virus-stimulated wells. The frequency of cytokine-producing cells was expressed as responding cells in 10^6^ PBMC.

### Swine fibroblast isolation and characterization as antigen presenting cells

Fibroblasts were obtained from the skin of survivor pigs following previously described protocols [63], with slight modifications. Briefly, 2 cm^2^ segments of skin were obtained from previously razed and chlorexidine-disinfected skin and immediately included in 0.5% Trypsin in PBS. After O/N incubation at 4°C, skin fragments were cultured in RPMI supplemented with 10% FCS. Primary fibroblast maintained their viability after multiple serial passages, retaining their capacity to present the SLAI-restricted 9-mer ASFV peptides to their autologous PBMCs in an IFNγ – ELISPOT assay. Briefly, fibroblasts were incubated *in vitro* for 1 h at 37°C with or without the specific peptides and after several washes, they were added as stimulators of their autologous PBMCs at 1∶10 ratio for 20 h and then the ELISPOT assay was continued as described above.

### Virus and challenge

All pigs were challenged intramuscularly with 10^4^ HAU_50_ of the highly virulent E75 ASFV isolate. Lethality was recorded as the gold standard marker of protection, since non-protected pigs should always succumb before day 9 post-infection due to the high lethal dose administered (>20 LD50s). Additionally, clinical signs of ASF (fever, anorexia, lethargy, shivering, cyanosis and recumbency) were monitored daily.

Blood samples were collected before and at different times after virus challenge for detection of viremia. The virus was titrated in porcine alveolar macrophages by a hemoadsorption assay. Briefly, serial dilutions of sera were incubated with 4×10^5^ porcine alveolar macrophages in each well. After 24 h porcine erythrocytes (whole blood diluted 1∶100 in PBS) were added to each well. After 12 h and every 24 h the plates were observed to follow the hemoadsorption effect. Titres were calculated and expressed as HAU_50_/ml.

Leukopenia due to ASFV infection was followed by counting the number of cells/ml of blood (1/100 diluted blood mixed 1∶1 with Turks solution) and also by flow cytometry using the diverse surface-cell markers described below.

### Flow cytometry

Surface PBMCs staining was performed as previously described [64], monoclonal antibodies against CD4 (74-12-4 clone), CD8 (76-2-11 clone), CD163 (2H12/BM clone) and SWC3 (BL1H7 clone) were used. Hybridoma supernatants (generously provided by J. Dominguez) were used diluted 1∶5 and secondary FITC conjugated anti-isotype antibodies (Vitro SA) were used at a dilution of 1∶100. Cell phenotypes were analyzed by flow cytometry (BD FACSaria I).

Additionally, surface expression of SLA I and SLA II molecules was also analyzed using primary isolated fibroblasts and monoclonal antibodies BL6H4 (anti-SLA I) and 1F12 (anti SLA II-DR), respectively, followed by an incubation with the corresponding FITC conjugated anti-isotype antibodies (Vitro SA).

### Statistical analysis

Survival data was subjected to the Logrank test of the Kaplan-Meier survival curves using the NCSS-PASS software.
